# Hearing thresholds in European sea bass (*Dicentrarchus labrax*): new insights into auditory sensitivity

**DOI:** 10.1007/s10695-025-01628-2

**Published:** 2026-01-04

**Authors:** Laura Rojas, Andreas Ruser, Johannes Baltzer, Clément Crouzet, Michael Schlachter, Joseph Schnitzler, Ursula Siebert, Maria Morell

**Affiliations:** 1 Institute for Terrestrial and Aquatic Wildlife Research (ITAW), University of Veterinary Medicine Hannover, Foundation, Werftstr. 6, 25761 Büsum, Germany; 2 ATC Hirtshals, BioMar A/S, Niels Juelsvej 38a, Hirtshals, Denmark; 3 Fraunhofer Research Institution for Individualized and Cell-Based Medical Engineering IMTE, Hafentoern 3, 25761 Büsum, Germany; 4 Department of Ecoscience, Aarhus University, 8000 Aarhus, Denmark

**Keywords:** AEP Audiogram, Auditory response thresholds, Auditory evoked potentials, Baseline hearing, European sea bass

## Abstract

Expanding our understanding of auditory sensitivity in fishes is essential not only for advancing sensory biology, but also for assessing the impact of underwater noise on marine life. However, knowledge remains limited for many ecologically and commercially relevant marine species, particularly auditory generalists like the European sea bass (*Dicentrarchus labrax*). In this study, we used auditory evoked potentials (AEPs) to measure hearing thresholds in 114 juvenile European sea bass across six frequencies (100–600 Hz), representing the largest dataset of its kind for this species. Our results revealed a U-shaped audiogram with highest sensitivity at 300 Hz (mean threshold: 116.8 ± 3.3 dB re 1 µPa), and thresholds up to 22 dB lower than previously reported. These findings suggest that *D. labrax* has higher auditory sensitivity than previously assumed. We also documented significant interindividual variability in hearing thresholds, particularly at lower frequencies, highlighting the importance of large sample sizes to capture natural variation. These baseline data provide a valuable reference for future studies on acoustic ecology, effects of noise exposure, and welfare optimization in aquaculture settings, and emphasize the need for further auditory research in marine fish species.

## Introduction

The study of hearing in fishes has developed significantly in recent decades due to its relevance in enhancing our scope of fundamental sensory biology, and addressing increasing concerns surrounding the ecological impacts of underwater noise (Popper & Hawkins 2019; Slabbekoorn et al. 2010). Hearing is an essential sense for fish, enabling them to gather information from their environment over long distances and from all directions, often beyond the capabilities of other senses such as vision or chemoreception (Fay 2009; Frisch 1938; Hawkins & Popper 2018). Because sound travels efficiently in water, it plays a key role in detecting prey and predators, navigating through habitats, locating spawning grounds, and, in some species, communicating with conspecifics (Ladich & Fay 2013; Radford et al. 2014). With increasing levels of anthropogenic underwater noise, there is also a growing need to better understand how different fish species detect and process sound. Despite advances in fish bioacoustics, hearing sensitivity data are still lacking for many ecologically and commercially important marine species (Ladich 2024; Popper et al. 2014).

Most studies of hearing in fish have focused on freshwater species, particularly those with anatomical specializations for enhanced auditory sensitivity, such as otophysan fishes (Ladich 2023) or other groups with auditory adaptations, such as Percopsiformes (Soares et al. 2016). In contrast, data on the hearing sensitivity of marine fishes remains comparatively scarce. This gap is particularly evident for species considered auditory generalists; i.e., species lacking known morphological specializations for sound detection, such as a connection between the swim bladder and the inner ear (Ladich & Popper 2004), despite their ecological relevance or importance in aquaculture.

The European sea bass (*Dicentrarchus labrax*) is a widely distributed euryhaline, teleost inhabiting coastal and estuarine waters of the eastern Atlantic Ocean and the Mediterranean Sea (Linnaeus 1758; Vázquez & Muñoz-Cueto 2014). It plays a key ecological role as both predator and prey in marine food webs and is also one of the most important species in Mediterranean aquaculture, with production steadily increasing in recent decades (Vandeputte et al. 2019; FAO 2022). Its high tolerance to salinity (0–40 ppt), temperature (2–32 °C), and environmental variation has made it a valuable model for physiological and behavioural studies (Pickett & Pawson 1994). However, despite its commercial and ecological value, relatively little is known about the sensory biology of *D. labrax*, particularly its hearing capabilities.

The hearing capabilities of the European sea bass are thought to be relatively limited in frequency range and sensitivity compared to auditory specialists. Although some behavioural studies have evaluated sea bass responses to noise exposure (e.g., changes in swimming, avoidance behaviour and startle responses, Kastelein et al. 2008, 2017; Neo et al. 2018; Vercauteren 2014), physiological data on their hearing capabilities are scarce. To date, one study has used auditory evoked potentials (AEPs, i.e. a neural response recorded from the surface of the head that reflects the synchronized activity of auditory pathways following sound stimulation) on a few individuals (*n* = 6) to assess hearing thresholds in this species, providing limited information on its auditory range and sensitivity (Lovell 2003, 2005). According to our research, no other AEP measurements have been reported in *D. labrax*.

The AEPs are a widely used electrophysiological technique for assessing hearing sensitivity in fishes (Brown et al. 2019; Casper & Mann 2009; Egner & Mann 2005; Ladich & Fay 2013; Lechner & Ladich 2011; Maurer et al. 2023). The method involves recording the neural responses elicited by sound stimuli using electrodes placed on or near the head of the animal (Kenyon et al. 1998). The AEPs represent the summed electrical activity of auditory pathways, primarily from the inner ear and brainstem, in response to acoustic signals. Because the technique is non-lethal, relatively fast, and suitable for a wide range of species and life stages, it has become a common tool in fish bioacoustics research (Kenyon et al. 1998; Ladich & Fay 2013). AEPs allow for the estimation of hearing thresholds across frequencies, providing data on auditory sensitivity that are not influenced by behavioural variability.

In addition to the general lack of hearing data for many fish species, few studies have examined interindividual variability in auditory thresholds reported differences within a species have often been linked to factors such as size, sex, or reproductive state (Chapman 1973; Ladich 2015; Maruska et al. 2012; Monroe et al. 2016; Schellart & Buwalda 1990; Tavolga & Wodinsky 1965). Exploring such variability is important for establishing reliable auditory baselines and a better understanding of how fishes may differ in their acoustic sensitivity. These data provide a reference point for future studies investigating the effects of environmental factors, including exposure to anthropogenic noise.

In this study, we recorded AEPs to determine hearing thresholds in a large number of European sea bass individuals, using tone pip stimuli across six low frequencies. This approach enabled us to detect short-latency neural responses to sound stimuli to establish auditory thresholds based on the presence or absence of these responses. By testing 114 individuals, we obtained a robust towards population-level estimate of hearing sensitivity and quantified the natural inter-individual variability within the species. These AEP audiograms provide the most comprehensive dataset to date on the auditory capabilities of *D. labrax* and provide an important reference point for future studies on fish bioacoustics, auditory physiology, and the potential impacts of underwater noise.

## Material and methods

### Fish and facilities

A total of 114 European sea bass (*D. labrax*) with a standard length of 13.5–17.6 cm (mean: 14.2 ± 1.21 cm) and a weight of 70–80 g was used in this study. All individuals originated from a captive population and were obtained from the aquaculture system of BioMar Group (Denmark). This large number of European sea bass was selected because these individuals were part of a subsequent study that required a large sample size.

BioMar Group facilities maintained the fish in tanks with a salinity of 33–34 ppm and a temperature of 23 °C. The fish were transported from Denmark to Germany by Freia Forellen Export A/S (EU TRACES exporter authorization no. 88441516) to ensure stable conditions throughout the route, including temperature and oxygen levels. The fish were subsequently placed in tanks at the Institute for Terrestrial and Aquatic Wildlife Research (ITAW), University of Veterinary Medicine Hannover, Germany, where the experiment was conducted.

Two plastic intermediate bulk containers (IBC) were customized for housing tanks (1.2 × 1.0 × 0.8). The tanks were filled with 600 L of seawater. Salinity and temperature were maintained at the same levels as the origin facilities and monitored with a CTD-Diver (Van Essen Instruments®). Every tank was equipped with a continuous external filter system (Tetra Aquarium external filter EX1500 Plus). A light cycle of 12:12 h was maintained and fish were fed twice per day with 2-mm pellets until apparent satiation (Ocean Nutriton®). Ammonium concentration levels (MQuant®) were analysed twice per week in all tanks. Ammonium levels were kept at 0 mg/L throughout the experiment, while the temperature and the salinity were kept at the same values as the origin facilities (23 °C and 33–34 ppm). The animals were housed following established guidelines, and all experiments were conducted with approval from the Ministry of Agriculture, Rural Areas, Europe and Consumer Protection (MLLEV), Schleswig–Holstein.

### Background noise measurements

The background noise at the original housing facility and the tanks from the laboratory at ITAW were measured to identify the sound pressure and sound energy levels (SPL & SEL) respectively. Recordings of 300 s were measured using an underwater sound recorder (SoundTrap ST 300 HF, OceanInstruments, New Zealand). Since the fish did not show swimming preferences, the recorder was placed in 6 equidistant positions distributed throughout the tanks, at a middle depth, following a designed grid. The background noise of the exposure chamber was also measured following the previously described methodology (see above). The recorder was positioned at the same location where the fish was placed to measure AEPs.

Once all the recordings were completed, the sound files were downloaded with SoundTrap Host Software version 4.0.19. A customized script in the R Studio software (R Core Team 2020) calculated SPLs and SELs. Spectral analyses were performed using the Welch method (1-s windows within 15-s recordings), and results are presented as power spectral density (PSD; dB re 1 µPa^2^ Hz).

### Auditory evoked potential (AEP) measurements

After the minimum of a one-week adaptation period, a randomly selected group of four individuals was transferred from the holding tank to the experimental tank. The fish were easily identifiable in the experimental tank by specific landmarks and were in the experimental tank and used as part of a subsequent study. The experimental setup consisted of a 1000 L Intermediate Bulk Container (IBC) customized plastic tank (1.2 × 1.2 × 1 m) filled with 800 L of water, equipped and maintaining the same environmental conditions (oxygen levels, temperature, and salinity).

The AEPs were performed sequentially on each of the four individuals. The AEPs were conducted in a customized container made of polyvinyl chloride (PVC) pipe (1.65 m length with 65 cm window in the middle and 38 cm high), closed at the sides, and oriented right-left to create a simple sound field. The PVC container was set up inside an anechoic chamber and was filled with the same water coming from the experimental tank at a height of 29 cm.

Prior to each test, a single fish was slightly anesthetized in a 70 mg/L tricaine methane sulfonate bath (MS-222) buffered with sodium bicarbonate to a pH of 7.0 (Chatain & Corrao 1992) until it lost the balance. Exposure time to the anaesthetic ranged from a few seconds to 3 min and its effect typically lasted about 6–9 min.

**Fig. 1:**
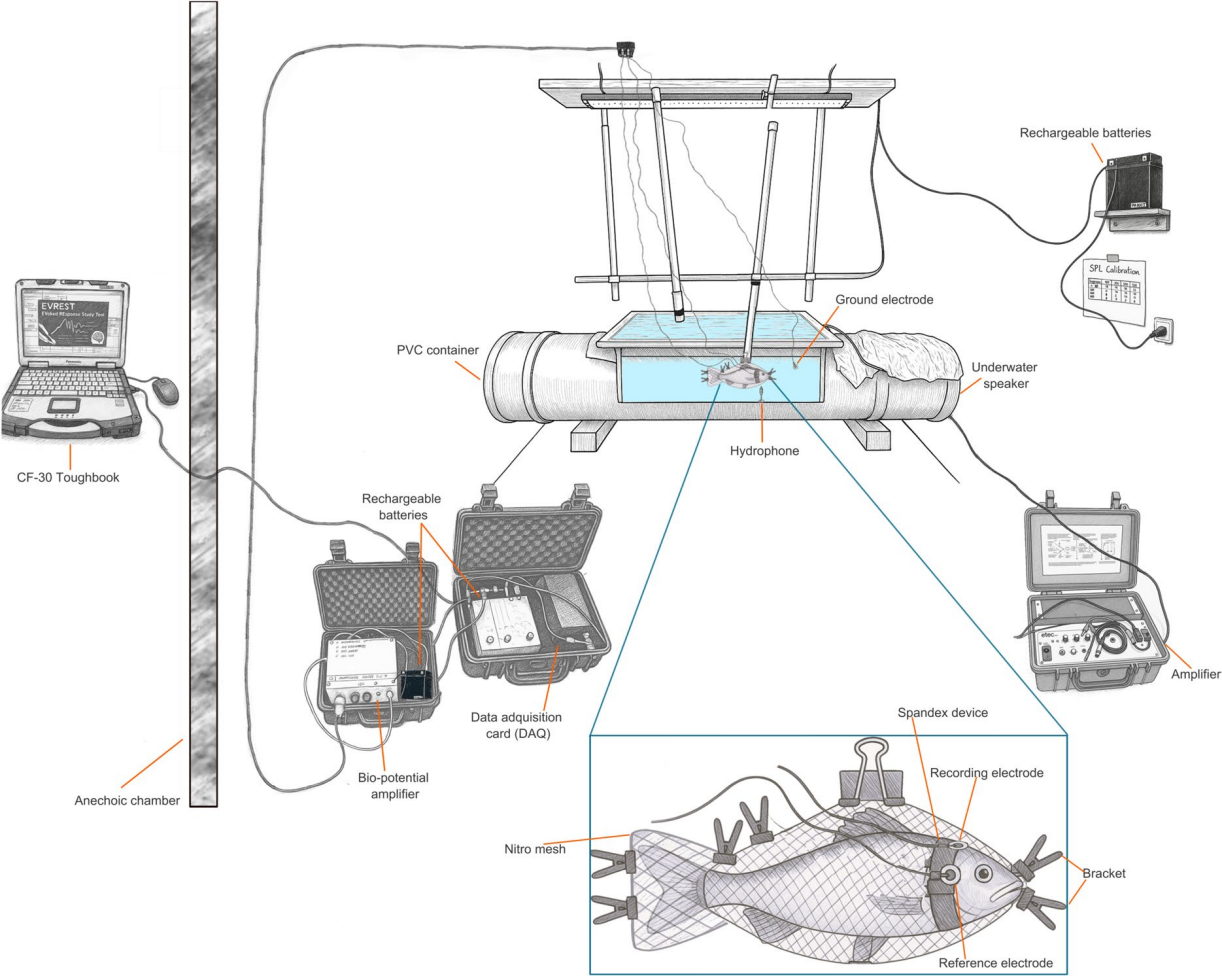
Experimental setup used for auditory evoked potential (AEP) measurements in European sea bass. A custom-made PVC tank was placed in an anechoic chamber and filled with seawater. Fish were fixed in a breathable mesh and suspended using lab brackets, with gold cup electrodes secured by a spandex band over the skull. Stimuli were generated via EVREST software and delivered through an underwater speaker, while a hydrophone and amplifier system recorded AEP responses under controlled acoustic conditions

The fish was fixed in a custom-made Nylon mesh, clamped and suspended from laboratory brackets, without additional anaesthesia within one centimetre of the hydrophone. The material allowed the fish to breathe while maintaining it in the correct position, restricting movement. Total AEP recording time per individual was 10–14 min with automated testing of the six predefined frequencies. The fish remained motionless throughout the AEP recordings due to the combined effect of mild anaesthesia and gentle restraint, and no spontaneous movements were observed during the measurements. Gold cup electrodes (TerniMed®) were used for recording the AEP signal. The electrodes were well placed by a custom-made device for each individual made by spandex fabric to hold the electrodes. The recording electrode ( +) was placed on the midline of the skull over the medulla region, the reference electrode ( −) was placed 1 cm posterior to the recording electrode, and a ground electrode was placed directly in the water close to the fish (Fig. 1).

Electrode impedance was maintained below 3 kΩ throughout the measurements to ensure proper signal quality. Signal amplification, filtering, and digitization were conducted following the methodology described by Maurer and collaborators (2023), using a bio-potential amplifier (CP511, GRASS Technologies Inc., West Warwick, RI, USA) and a data acquisition card (NI USB 6251, National Instruments, Inc., TX, USA). Once the electrodes were in place, the fish was carefully positioned in the testing container to maintain stable recording conditions.

A preliminary trial was conducted using eight fish to establish the optimal test frequencies for this species under our experimental setup. Auditory response thresholds were assessed at 100, 200, 300, 400, 500, and 600 Hz. The acoustic stimuli were generated digitally via a CF-30 Toughbook (Panasonic Connect Europe GmbH, Wiesbaden, Germany) and subsequently converted to analogue signals using a USB multifunction data acquisition card (NI USB 6251, National Instruments, Inc., TX, USA). The signals were updated at a 1 MHz rate with a 16-bit resolution and processed through a bandpass filter (100–250,000 Hz, 24 dB/decade, Krohn Hite, Inc., Brockton, MA, USA) before transmission via an underwater speaker (UW-30, Lubell Labs, Inc., Columbus, OH, USA), positioned on the right side of the experimental container. The stimuli comprised 1024 repetitions in alternating 1–0-1 tone pip sequences, with a sweep duration of 21 ms for each frequency. Each pip contained two cycles of the waveform, shaped by a cosine envelope. The delivery of sound stimuli and the recording of auditory evoked potentials (AEPs) were carried out using the Evoked Response Study Tool (EVREST) software (Finneran 2009; Finneran et al. 2008).

The initial sound amplitude was set at 110 dB re 1 µPa for all measurements. The SPLs were progressively increased in 10 dB increments until the first detectable AEP response was observed. SPL was then reduced in 5 dB steps to refine the detection threshold. Thresholds were defined as the x-intercept of a linear regression of Fsp vs SPL across ≥ 5 SPL levels (2.5–10 dB steps), with R^2^ ≥ 0.8 required for acceptance; otherwise, the series was repeated. After a fish had been tested, it was put back into the experimental tank and used for a subsequent study. SPL calibration was performed before each trial by averaging 1024 stimuli at 121 dB re 1 µPa, using a TC4013 hydrophone (RESON, Teledyne Marine, USA) positioned at the fish’s location during stimulus presentation. Calibration checks were conducted before and after trials to detect potential variations. The recorded stimulus signals were pre-amplified by 20 dB, bandpass filtered from 1 to 180,000 Hz (B1501 amplifier, Etec, Denmark), and digitized at 500,000 Hz with a 16-bit DAQ-card (NI USB 6251, National Instruments, Inc., USA). EVREST software controlled SPL adjustments and stimulus presentation throughout the procedure. To confirm that AEP waveforms represented true neural responses and not electrical artefacts, control recordings were performed on recently euthanized fish, which produced no repeatable responses.

### Auditory evoked potential analysis

The AEP data were processed and analysed for signal detection following the methodology described by Maurer et al. (2023), and based on the signal detection methods described by Don et al. (1984) and Elberling and Don (1984). The signals were filtered and analysed using a statistical F-test to compare the AEP with background noise. Significant oscillations indicated sound detection (“hit”), while their absence was considered a “miss.” Response thresholds were determined by the SPL corresponding to the lowest “hit” and highest “miss”. Furthermore, two trained assessors reviewed all responses to ensure accuracy.

After obtaining the AEP measurements, auditory thresholds were statistically analysed to evaluate both frequency-dependent differences and interindividual variability. Differences in median SPL thresholds across frequencies were tested using a Kruskal–Wallis test, followed by Mann–Whitney–Wilcoxon post hoc pairwise comparisons with Holm’s stepwise correction. Variability among individuals was assessed using a Brown–Forsythe (Levene’s) test with the median as the centre, and descriptive measures of dispersion (SD, IQR, MAD) were calculated for each frequency. In addition, to examine whether individual sensitivity was consistent across frequencies, Person’s correlation coefficient was calculated between thresholds at each pair of frequencies within individuals. The intraclass correlation coefficient (ICC, McGraw & Wong 1996) was performed to quantify the proportion of variance attributable to individual differences versus measurement error. All analyses were conducted in R 4.1.19 (R Core Team 2020).

## Results

### Background noise

**Fig. 2:**
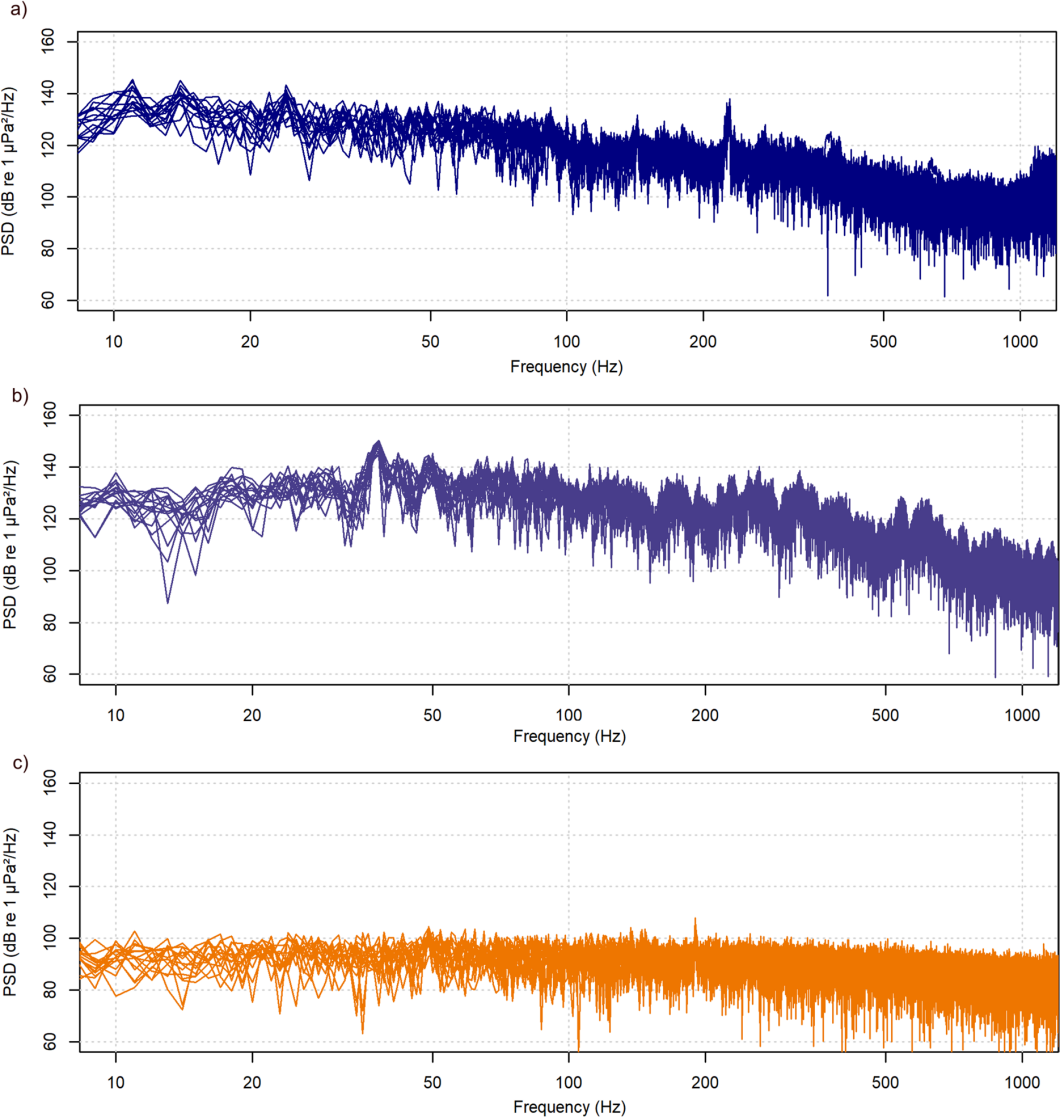
Power spectral density (PSD, Welch method) of background noise recorded for European sea bass (*Dicentrarchus labrax*) in (**a**) the BioMar housing tank at the origin facilities, **b **the ITAW holding and experimental tanks, and (**c**) the PVC container used for AEP recordings. Each line represents the spectrum of a 1-s segment within a 15-s recording (60–75 s). Occasional peaks correspond to fish swimming close to the hydrophone, but overall noise conditions were stable. The spectral profiles of the BioMar and ITAW tanks were highly comparable, indicating that fish experienced similar acoustic environments before and during the experiments, while lower background levels were observed in the PVC container used for AEP measurements

The background SPLs of the European sea bass holding tank at the housing facilities had a range of 120–128 dB re 1 µPa. In addition, the single SEL showed a fluctuation between 110 and 116 dB re 1 µPa^2^s (Fig. 2a). At ITAW facilities, the SPL was calculated in a range of 123–126 dB re 1 µPa, while the single SEL showed a fluctuation of only 2 dBs (113–115 dB re 1 µPa^2^s (Fig. 2b). Occasional peaks were caused by fish swimming near the hydrophone, but these did not affect the overall stability of the spectra.

The SPLs in the PVC container where the AEPs were measured were 91–110 dB re 1 µPa. The single SEL had a minimum variation of 0.4 dB with values between 80.8 and 81.2 dB re 1 µPa^2^s, with consistent spectra across the 15-s segment (Fig. 2c).

### Novel lower AEP thresholds measured in sea bass

**Fig. 3:**
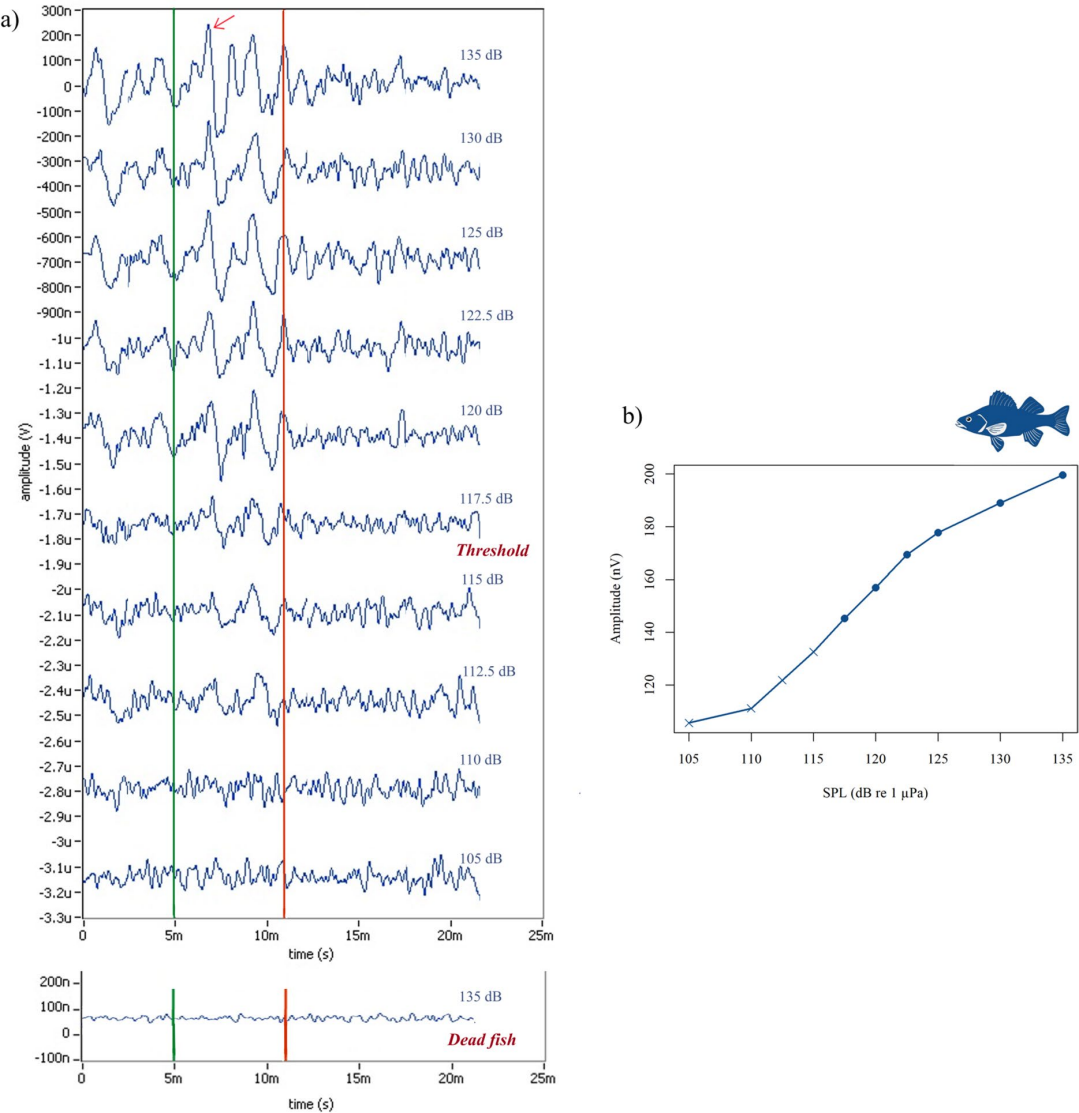
Example of auditory evoked potential (AEP) threshold estimation for a single European sea bass at 200 Hz. **a **Average AEP waveforms recorded from a European sea bass in response to a 200-Hz tone pip stimulus, presented at sound pressure levels (SPLs) ranging from 105 to 135 dB re 1 µPa SPL, using variable step sizes of 2.5, 5, or 10 dB re 1 µPa SPL. Each trace represents the average of 1024 stimulus repetitions. The AEP waveforms increased in amplitude as SPL increased. The bottom panel represents a recording from a dead individual, showing no evoked response and confirming the physiological origin of the signals. **b **Threshold estimation was performed using the single-point F-test (Fsp), a time-domain technique implemented in EVREST software. The Fsp values were plotted as a function of SPL, and the x-intercept of the best-fit regression line was used to estimate the statistical AEP threshold. For this individual, the Fsp-derived threshold at 200 Hz was 116.28 dB re 1 µPa

The AEPs recorded from European sea bass showed consistent waveform morphology across different SPLs. At suprathreshold SPLs, responses were characterized by a series of alternating negative and positive peaks, superimposed on a broad slow negative deflection, often followed by a return to baseline or a shallow positive wave. As SPLs decreased, the overall amplitude of the waveforms was reduced, and peak clarity diminished, though identifiable components remained close to threshold. In contrast, recordings from non-responsive (dead) individuals under the same conditions showed no structured or repeatable waveforms, confirming the physiological origin of the AEPs observed in live fish (Fig. 3).

**Fig. 4:**
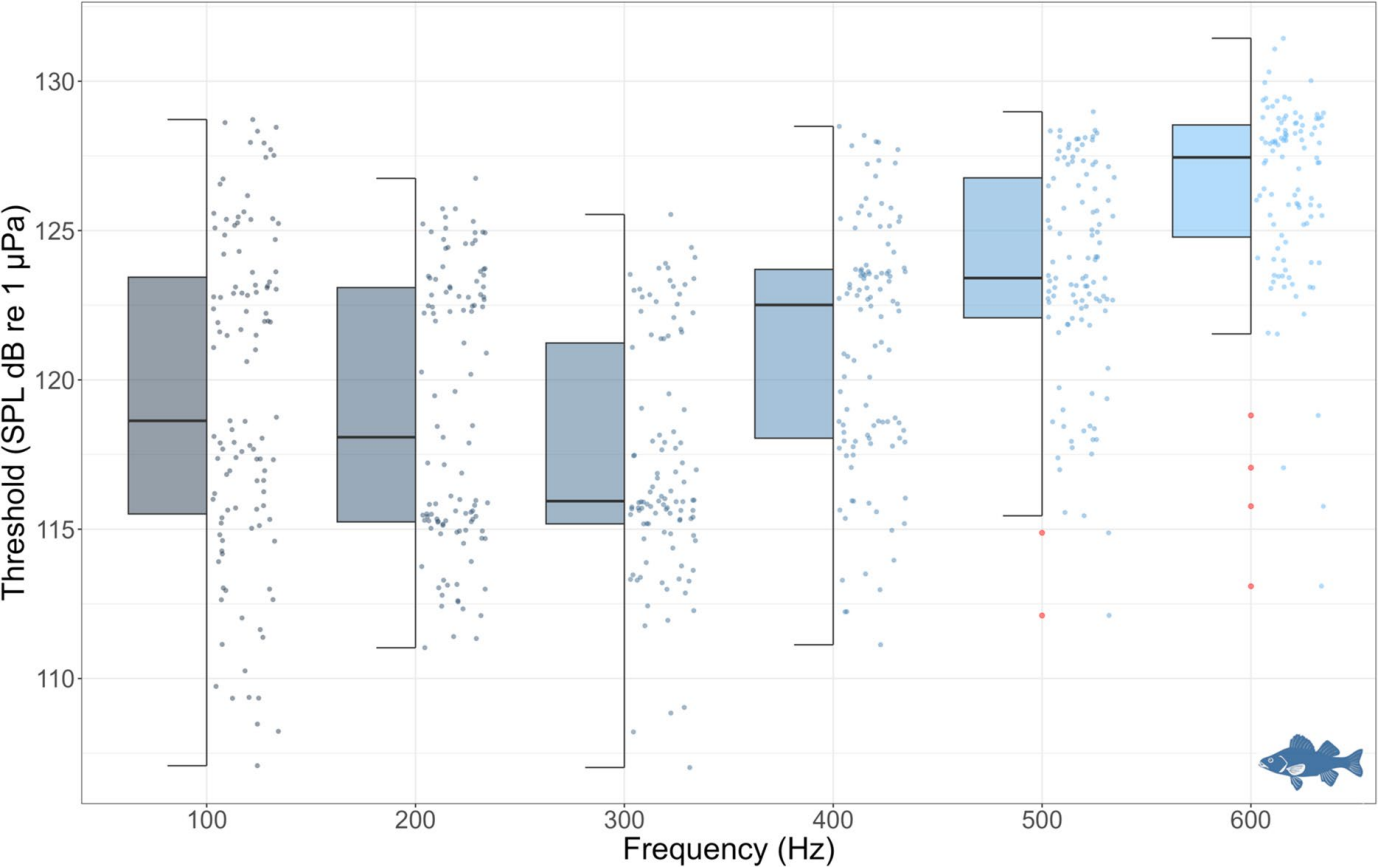
Boxplot AEP audiogram of European sea bass based on auditory evoked potential (AEP) thresholds at six frequencies (100–600 Hz). Each boxplot shows the median, interquartile range, full data spread, and statistically defined outliers (in red). Thresholds represent the sound pressure level (SPL) at which a detectable AEP response was recorded for each individual (*n* = 114). Lower frequency-dependent variation in hearing thresholds indicate greater auditory sensitivity to acoustic stimuli

Audiograms were generated for six stimulus frequencies (100, 200, 300, 400, 500, and 600 Hz) using tone pip stimuli. Although 114 individuals were tested in total, slight variations in sample size per frequency occurred due to movement artifacts or incomplete recordings (n ranged from 107 to 114). The lowest individual threshold values were found at 300 Hz (107 dB re 1 µPa), and adjacent frequencies (200 Hz = 111 dB re 1 µPa; 400 Hz = 111.1 dB re 1 µPa), indicating a range of greatest auditory sensitivity centred around 300 Hz. Mean thresholds increased at the frequency extremes, reaching 119.5 dB re 1 µPa at 100 Hz and 127.4 dB re 1 µPa at 600 Hz (Table 1). Maximum thresholds ranged from 125.5 to 131.4 dB re 1 µPa. Standard errors of the mean thresholds ranged from 3.1 to 4.1 dB. Despite interindividual variability, the overall AEP-derived audiogram showed a consistent U-shaped pattern of sensitivity across frequencies (Fig. 4).

**Table 1:** Mean auditory thresholds (dB re 1 µPa) of European sea bass (*Dicentrarchus labrax*) at six different frequencies

Frequency Hz	Mean SPL threshold dB re 1 µPa	SPL Minimum threshold value dB re 1 µPa	SPL Maximum threshold value dB re 1 µPa	Standard error dB	*n*
100	119.4	110.0	128.7	3.5	111
200	118.9	111.0	126.7	3.2	114
300	117.2	107.0	125.5	3.3	107
400	121.2	111.1	128.4	4.1	108
500	123.6	112.1	129.9	3.2	111
600	126.4	114.0	131.4	3.1	112

**Fig. 5:**
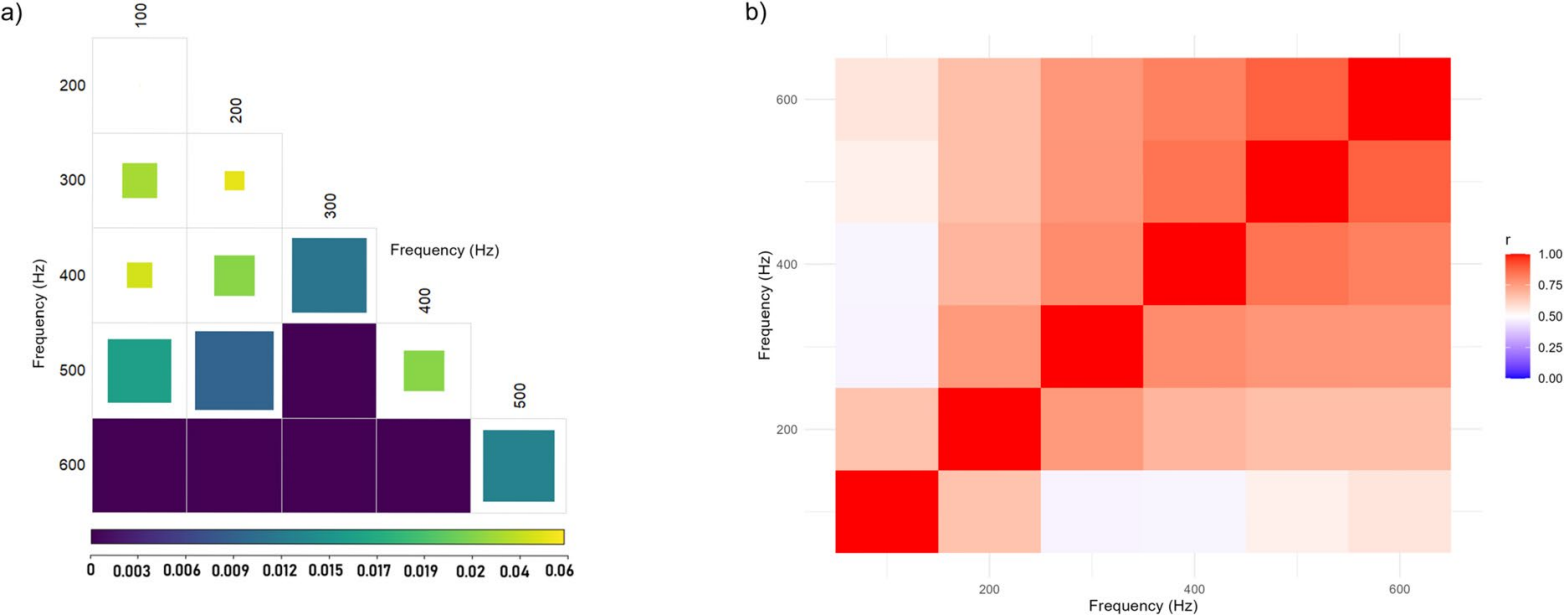
**a **Heatmap showing pairwise *p*-values for differences in AEP threshold medians across frequencies. The analysis was based on Kruskal–Wallis tests with post hoc pairwise comparisons; darker shades indicate lower *p*-values. Significant differences (*p* < 0.05) reflect shifts in median auditory thresholds between frequency pairs. **b **Heatmap showing Pearson correlation coefficients between auditory thresholds across frequencies at the individual level. Colours indicate the strength of the correlation (blue = low correlation; red = strong positive correlation). Correlation values ranged from *r* = 0.47 to 0.89, indicating that thresholds at different frequencies tended to covary within individuals

Significant differences in auditory thresholds across frequencies were found (H = 240.25, df = 5, p < 2.2e-16), indicating frequency-dependent variation in SPL values. Post hoc pairwise comparisons confirmed that the most pronounced differences occurred between 100 and 500 Hz (*p* < 6.2e-08), 100 Hz and 600 Hz (*p* < 2e-16), and 200 Hz and 600 Hz (*p* < 2e-16). Additional, though less marked, differences were found between 200 and 300 Hz (*p* = 0.0078) and between 300 and 400 Hz (*p* = 0.0268) (Fig. 5).

Interindividual variability in thresholds was greatest at lower frequencies, particularly at 100 Hz (SD = 5.48 dB, IQR = 7.93, MAD = 6.35), and gradually decreased toward higher frequencies, reaching its lowest values at 600 Hz (SD = 3.07 dB, IQR = 3.76, MAD = 2.22). The Brown–Forsythe (Levene’s) test confirmed significant heterogeneity of variance among frequencies, F (5, 642) = 14.42, *p* < 2.1 × 10⁻^13^. This pattern demonstrates a frequency-dependent distribution of interindividual variability, with lower frequencies showing the strongest dispersion across individuals. Thresholds were positively correlated across frequencies (Pearson’s *r* = 0.47–0.89; Fig. 5b), indicating that individuals with lower thresholds at one frequency tended to show lower thresholds at others. The intraclass correlation coefficient (ICC = 0.48 for single measures; ICC = 0.93 for average measures) further confirmed high within-individual consistency in hearing sensitivity.

## Discussion

This study provides the most comprehensive characterization of hearing sensitivity in European sea bass (*Dicentrarchus labrax*) to date, based on AEP thresholds recorded from 114 individuals. The results showed a broader hearing range and lower thresholds than previously reported (Lovell 2003, 2005), with maximum sensitivity at 300 Hz. The U-shaped audiogram and waveform morphology are consistent with patterns observed in other teleost fishes (Bullock & Corwin 1979; Nieder et al. 2023), and comparable to the AEP response profiles reported in other perciform species (Kenyon et al. 1998).

**Fig. 6:**
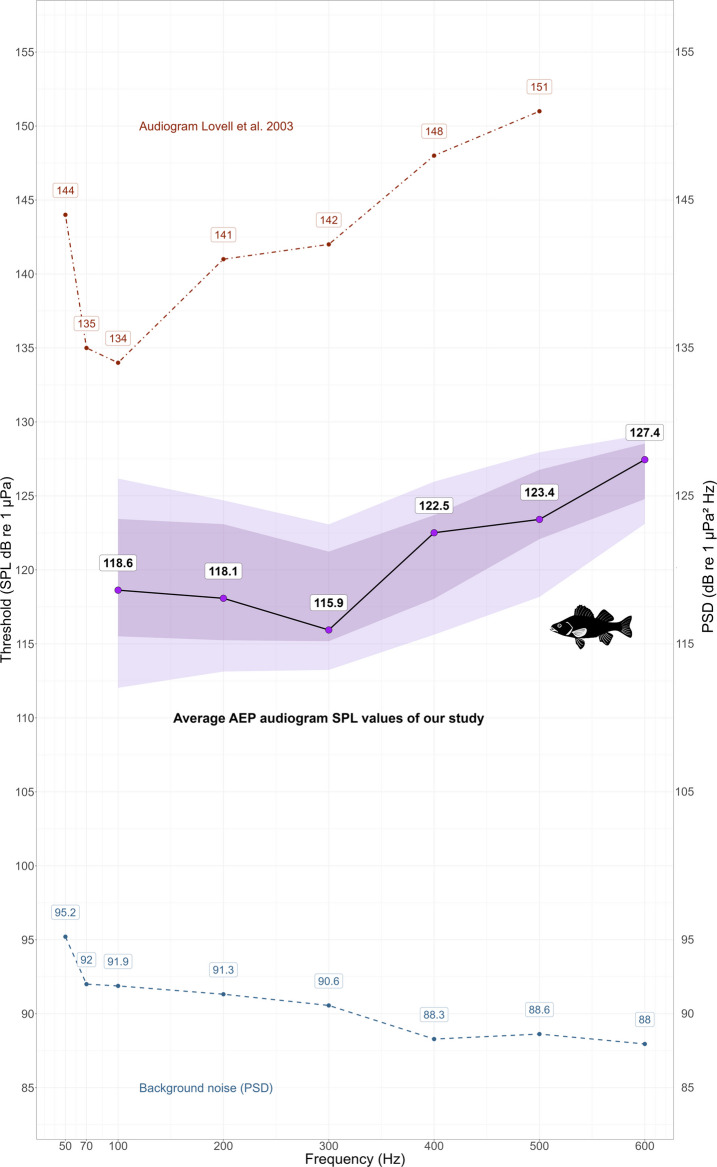
Auditory evoked potential measurements of the European sea bass (*Dicentrarchus labrax*) obtained in the present study. The solid black line represents the median auditory threshold (SPL in dB re 1 µPa) recorded for each tested frequency. Shaded areas indicate variability in the data: the dark purple band represents the interquartile range (25th–75th percentiles), while the light purple band spans the 10th to 90th percentiles. The dashed red line shows the AEP thresholds reported by Lovell (2003), assumed to represent mean thresholds following standard AEP procedures (Kenyon et al. 1998; Ladich & Fay 2013). The dashed blue line indicates background noise levels expressed as power spectral density (PSD; dB re 1 µPa^2^/Hz), plotted using a secondary y-axis on the right. Together, these methodological improvements led to a more detailed audiogram and augmented accurate estimations of hearing thresholds. However, it is important to note that auditory thresholds obtained using AEP methods are typically higher than those derived from behavioural techniques. As highlighted by Popper and Fay (2011), AEP-based measurements may underestimate true auditory sensitivity due to differences in the type of neural response recorded and the nature of stimulus presentation. Interestingly, our AEP thresholds align closely with the startle response thresholds reported by Kastelein et al. (2008), who employed a behavioural approach in the same species. The present study and Kastelein et al. (2008) identified similar thresholds in the low-frequency range (100–200 Hz), suggesting that *D. labrax* may possess greater auditory sensitivity than previously thought. This convergence of independent methodologies offers a valuable cross-validation of our results and supports the robustness of the thresholds described. These findings carry important implications for understanding the acoustic ecology of the species and its vulnerability to anthropogenic noise in natural environments

Compared to the only previously published AEP audiogram for this species (Lovell 2003, 2005), our data indicates that sea bass have a higher hearing sensitivity than previously measured. Lovell reported thresholds between 134 and 151 dB re 1 µPa for 100–500 Hz, with the lowest threshold at 100 Hz (134 dB). In contrast, our results show lower mean thresholds, ranging from 116.8 dB at 300 Hz to 127.4 dB at 600 Hz. At 300 Hz, the mean threshold was 116.81 ± 3.3 dB re 1 µPa, more than 17 dB lower than Lovell’s value for the same frequency (142 dB). At 200 Hz, the difference reached 22 dB (118.89 dB vs. 141 dB) (Fig. 6). The differences observed between the two studies may be influenced by various factors, such as a larger sample size in the present study (*n* = 114 vs. *n* = 6), modifications in signal processing and acoustic calibration, adjustments in ambient noise control (Ladich and Wysocki 2009; Wysocki and Ladich 2003, 2005), and the use of alternative threshold detection criteria, such as the single-point F-test (Maurer et al. 2023).

Although the present study provides new and detailed insights into the auditory sensitivity of European sea bass using AEPs, particle motion was not measured in our study. As an auditory generalist, the European sea detects sound primarily through particle motion, particularly at low frequencies (Nedelec et al. 2016; Popper & Fay 2011). In small experimental tanks, however, the acoustic field is complex and pressure and particle motion cannot be completely separated (Parvulescu 1967; Radford et al. 2012; Rogers et al. 2016). Campbell et al. (2019) further highlighted that confined tanks introduce artificial reverberation and standing waves, making it technically challenging to quantify particle motion accurately. These factors can introduce spatial and spectral distortions that influence both the delivery of the stimulus and the neural responses. Consequently, the AEP thresholds reported here in terms of sound pressure level (dB re 1 µPa) should be interpreted as reflecting the fish’s sensitivity to particle motion, rather than to the pressure component per se. This methodological limitation is widely recognized in AEP studies on auditory generalists and should be considered when using these data as a baseline for future comparative or applied research. Despite this constrain, AEP remains a valuable and widely accepted method for assessing auditory thresholds in fishes (Casper & Mann 2009; Egner & Mann 2005; Lechner & Ladich 2011), and our data provides useful baseline information for this species. Future research incorporating direct measurements of particle motion using accelerometers or shaker tables would enhance understanding of the underlying hearing mechanism in auditory generalists such as the European sea bass.

In addition, another crucial factor influencing the accuracy of auditory threshold estimation is the background noise. Elevated ambient noise levels can mask acoustic stimuli and artificially raise hearing thresholds, especially when noise overlaps with the most sensitive frequency range of species (Amoser & Ladich 2005; Fay 1974). This effect is well documented in teleost species, where increased environmental or tank noise resulted in threshold shifts (Buerkle 1968; Scholz & Ladich 2006; Wysocki & Ladich 2005). Therefore, it is essential to carefully monitor and quantify background acoustic conditions to ensure that threshold estimates truly reflect hearing abilities of fish and are not mistaken by masking.

In this study, background noise was systematically monitored across all experimental environments to ensure it did not confound threshold estimations. In the sea bass holding tanks, SPLs ranged from 120 to 128 dB re 1 µPa, with single-event sound levels (SELs) between 110 and 116 dB re 1 µPa^2^s. Similar levels were observed in the ITAW facility tanks. In contrast, the dedicated AEP tank (Pipe PVC container) showed lower acoustic levels, with SPLs ranging from 90.5 to 109.6 dB re 1 µPa, SELs between 80.8 and 81.2 dB re 1 µPa^2^s, well below thresholds shown to induce masking in other species. Spectral analyses revealed that most background noise energy was concentrated below 300 Hz, with dominant components under 100 Hz, frequencies often associated with electronic interference from setups (Wysocki & Ladich 2005). These spectral components lie outside the range of optimal auditory sensitivity in *D. labrax* (Lovell 2003 and our study) and remained below the thresholds measured in this study.

Beyond experimental contexts, background noise is also a critical concern in applied settings such as aquaculture. Chronic exposure to elevated noise levels in recirculating systems and hatcheries has been shown to impact fish physiology, behaviour, and welfare (Hang et al. 2021; Radford & Slater 2019). For an economically important species like the European sea bass, establishing baseline hearing sensitivity to sound pressure is fundamental for defining acoustic conditions that minimize stress and optimize growth. The thresholds reported in this study provide essential reference values to guide the design of quieter rearing systems and inform mitigation strategies aimed at reducing acoustic disturbance. Such efforts may contribute not only to improved welfare, but also to enhance production outcomes in intensive aquaculture operations (Sholes & Coffin 2024).

Finally, this study also revealed significant interindividual variability in AEP thresholds across frequencies, with the greatest dispersion observed at the lower frequencies (100 and 200 Hz). These differences may not be attributed to age or size, as all individuals were juveniles of similar morphometrics and developmental stage. While sexual dimorphism has been reported in *D. labrax* (Gorshkov et al. 1999), it is unlikely to have influenced our results given the early life stage of the fish and the absence of externally distinguishable sex characteristics. Correlation analyses among frequencies revealed moderate to strong positive relationships (r = 0.47–0.89; Fig. 5b), indicating that individuals with lower thresholds at one frequency tended to show lower thresholds across others. The intraclass correlation coefficient (ICC = 0.48 for single measures; ICC = 0.92 for average measures) further confirmed high within-individual consistency in hearing sensitivity. Together, these results demonstrate that auditory sensitivity in *D. labrax* represents a stable individual trait rather than random variation or proximity to detection limits. Early work by Tavolga & Wodinsky (1965) also reported pronounced interindividual variability in fish hearing, particularly at low frequencies, which they attributed partly to functional or behavioural factors such as learning or shifts in detection strategies (e.g., between the swim bladder–inner ear and lateral-line systems) rather than to age or morphological variation. Schellart & Buwalda (1990) later emphasized that structural and physiological differences among individuals, such as otolith mass or orientation, swim bladder, inner ear coupling, and neural synchrony, can likewise shape auditory performance. The individual profiles found here are consistent with these interpretations, suggesting that stable intrinsic differences underlie auditory sensitivity in *D. labrax*, particularly at frequencies dominated by particle-motion detection. The large sample size used in this study (*n* = 114) provided the statistical resolution needed to detect this natural variation, offering a more representative estimate of the auditory capabilities in the European sea bass. Understanding the degree of variation in hearing sensitivity among individuals of the same species is essential for identifying patterns that may be biologically relevant in future studies, including ontogenetic changes, environmental influences, or stress responses.

## Conclusions

In summary, this study provides the most detailed characterization to date of hearing sensitivity in European sea bass (*Dicentrarchus labrax)*, based on a large, homogeneous sample (*n* = 114) and a non-invasive AEP protocol. By combining surface electrodes, custom-fitted holders, and carefully monitored acoustic conditions, we obtained reliable auditory thresholds across a broad frequency range. Our results reveal higher sensitivity than previously reported, particularly around 300 Hz, and extend the known hearing range of the species up to 600 Hz. Thus, we obtained a more advanced description of auditory sensitivity and addressed an important gap in fish bioacoustics.

Furthermore, our experimental design enabled the first systematic assessment of interindividual variability in auditory thresholds in this species. This variability, often underexplored in fish hearing studies, highlights the biological relevance of using larger sample sizes to identify natural sensory diversity when establishing population-level baselines. While we acknowledge the limitations of not quantifying particle motion, the consistency of our sound pressure-based thresholds under well-characterized acoustic conditions provides a trustworthy baseline for further research.

The baseline data presented offers a valuable reference for future studies on noise exposure, auditory plasticity, and the ecological and physiological implications of acoustic disturbance. These findings also have practical relevance for aquaculture, where understanding species-specific hearing capacities can contribute to noise mitigation strategies and improve welfare conditions.

Finally, our study demonstrates that hearing in *D. labrax* is not yet fully characterized, and that critical aspects of auditory function remain to be explored, not only in this species, but in many others of ecological and economic importance. Establishing detailed auditory baselines is therefore essential for interpreting future changes in hearing and for guiding both conservation and aquaculture practices.

## Data Availability

The raw data supporting the conclusions of this article will be made available by the authors, without undue reservation.
